# Transitioning Towards Quality-of-Care Assessment in Orthotics and Prosthetics Practice

**DOI:** 10.1016/j.arrct.2026.100586

**Published:** 2026-01-22

**Authors:** Fanny Schultea, Andreas Kannenberg, Shane R. Wurdeman, Allen W. Heinemann, M.G. Finco

**Affiliations:** a The Orthotics and Prosthetics Foundation for Education and Research, Tomball, TX; b Ottobock Healthcare LP, Austin, TX; c Hanger Clinic, Austin, TX.; d Shirley Ryan AbilityLab, Northwestern University, Chicago, IL; e Texas Christian University, Fort Worth, TX

**Keywords:** Artificial limbs, Orthotic devices, Quality improvement, Quality of health care, Rehabilitation, Value-based health care

## Abstract

Quality of care is fundamental for achieving optimal patient outcomes, enhancing patient experiences, and engaging in improvement practices. Quality of care comprises 6 domains: safety, effectiveness, patient-centeredness, timeliness, efficiency, and equity. Despite robust frameworks guiding quality measurement in other health care fields, orthotics and prosthetics (O&P) rehabilitation lags in adopting these practices. As quality-of-care measures become more prevalent, particularly as the Centers for Medicare and Medicaid Services plans to shift to value-based care by 2030, the urgency to establish measures in O&P increases. Establishing specific evidence-based quality measures in O&P empowers the profession to define appropriate standards, helping to ensure that care remains tailored to patients’ needs and produces optimal outcomes—while minimizing the risk of inappropriate, externally imposed benchmarks. Several key groups across the care continuum are highlighted in this article, because they will be responsible for transitioning toward quality-of-care assessment in O&P care: leaders in O&P organizations, researchers, manufacturers, clinic owners, clinicians, patients, and educators. When each group fulfills its responsibilities, it strengthens overall quality and enhances the value of care provided. This special communication highlights the imperative of incorporating quality-of-care measurement into O&P practice across each key group and calls for research to define meaningful measures, establish standardized indicators, and implement systems for quality improvement. This article provides a comprehensive perspective to inform and educate each key group in quality-of-care concepts to foster improvement efforts in O&P.

## The importance of measuring quality of care in orthotics and prosthetics

Quality-of-care measurement and reporting can enhance patient outcomes, ensure the sustainability of orthotics and prosthetics (O&P) rehabilitation, and allow more patients to benefit from O&P rehabilitation. Implementing quality-of-care measures and reporting practices that are specific to O&P also has the potential to improve care delivery through a reimbursement model based on quality metrics.

Professions such as nursing, occupational therapy (OT), and physical therapy (PT) have contributed to literature that identifies and implements quality-of-care measures. Although OT and PT have published research on quality of care since the 1970s,^1^, ^2^, ^3^, ^4^ literature specifically addressing quality in O&P is limited. To the authors’ knowledge, only 6 studies have been published in the last 5 years, focusing on custom ankle-foot orthoses, upper-limb prostheses,^5^, ^6^, ^7^, ^8^ and service quality.^9^^,^^10^ Two of these studies identified quality-of-care priorities^5^^,^^6^ in the use of custom ankle-foot orthoses, which provide foundational research for determining specific and meaningful quality-of-care measures. Another study identified instruments to assess the quality of care for custom ankle-foot orthoses interventions.^7^ The study on upper-limb prosthetic interventions assessed both quality of care and patient satisfaction in Veterans with upper-limb loss.^8^ One service-quality study examined patient satisfaction and experience across O&P care in 14 US sites in 2025,^9^ and the other highlighted a lack of service-quality data across orthotic care in the United Kingdom.^10^

Although these studies each report aspects of quality of care, to our knowledge, no article has defined quality of care for O&P populations with actionable items to facilitate implementation into O&P practice, yet. As value-based care becomes more widespread, conducting targeted and comprehensive quality-of-care research in O&P will better position the profession to adapt to changing health care standards. Therefore, this special communication defines quality of care, its relationship to the value of care, and guides key groups in O&P to support the establishment of O&P-specific measures and indicators, the development of quality programs, and their successful implementation in O&P practice.

## Defining quality in health care

### Frameworks, domains, and resources

The World Health Organization defines quality of care as “the degree to which services for individuals and populations increase the likelihood of desired health outcomes.”^11^ This concept, initially established by Donabedian’s^12^ model in 1966, categorizes quality measures across 3 interrelated dimensions (1) structure (eg, clinic facilities, staff training, and clinic, insurance, and government policies); (2) process (eg, methods of care, and delivery and documentation); and (3) outcomes (eg, patient satisfaction, morbidity and mortality, and changes in health status). Although emphasis has been placed on objective clinical quality measures (eg, morbidity and mortality and rates of hospital readmission), there has been a shift toward incorporating patient-reported measures such as patient satisfaction with services.^13^ However, these dimensions do not cover all quality-of-care domains or recently proposed domains (eg, eco-friendly and transparency).^14^

**Fig 1:**
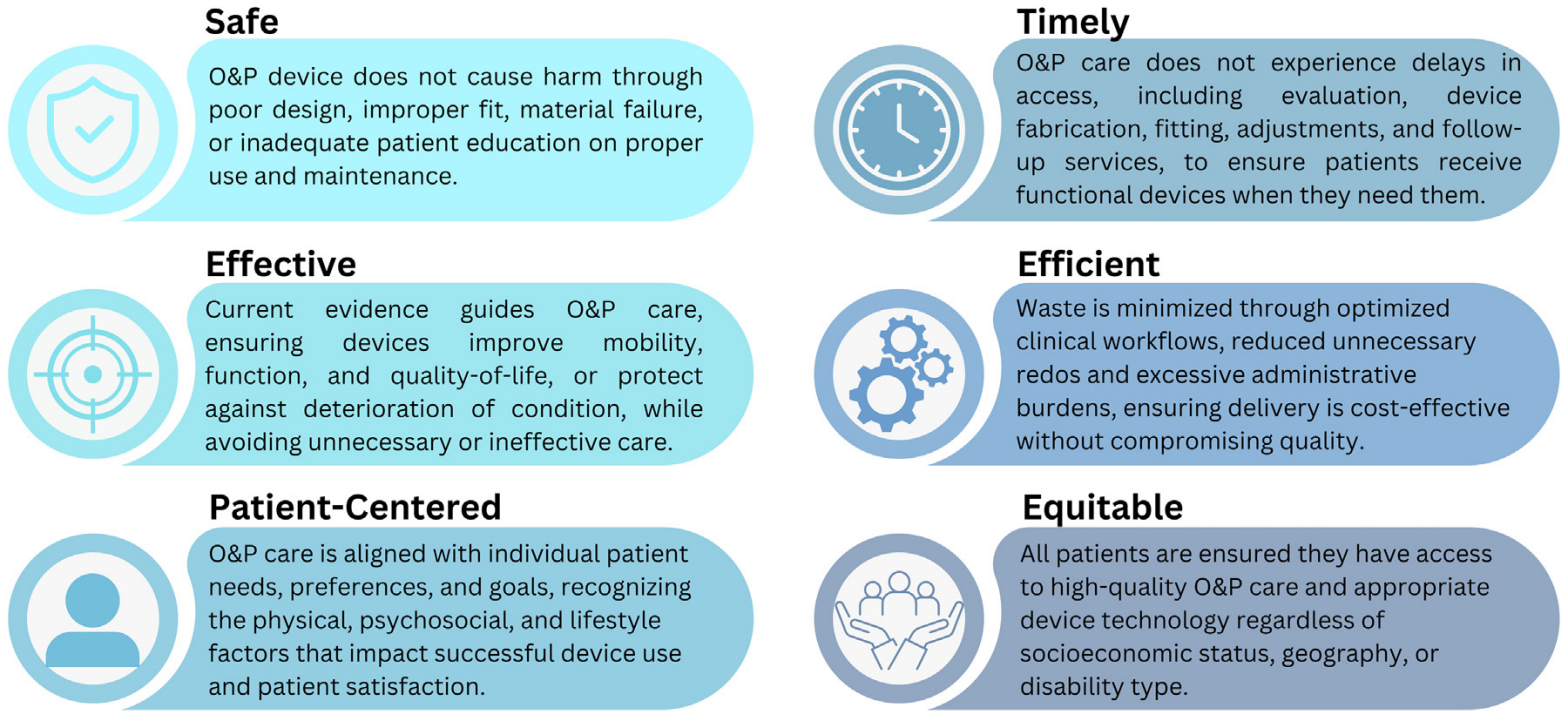
The IOM’s 6 quality-of-care domains adapted to O&P care.

The Institute of Medicine (IOM) proposed a framework for the development of quality-of-care measures in 2001.^15^ This framework outlines 6 domains of quality measurement in a health care system: safe, effective, patient-centered, timely, efficient, and equitable.^11^^,^^15^^,^^16^ IOM subsequently proposed measurement recommendations for these domains in 2005.^17^^,^^18^ Figure 1 depicts the 6 domains with definitions and examples that are relevant to O&P.

A complementary model that is used in quality of health care and quality improvement work is the Triple Aim, which was proposed by the Institute for Healthcare Improvement in 2008 and encompasses 3 aims: patient population health, patient experience of care, and patient cost of care per capita.^19^ In 2022, the Quintuple aim framework was proposed to include the advancement of health equity and the well-being of the health care workforce.^20^ Although these characteristics represent an ideal health care system, this framework offers guidance to improve population health and well-being, to advance and sustain better health outcomes, and maximize the value of the care provided.^21^ In the last decade, specialized frameworks, more specific to rehabilitation settings or pathologies, have been developed.^22^, ^23^, ^24^, ^25^

Several organizations provide resources to guide quality-of-care metric development and selection (table 1).^26^, ^27^, ^28^, ^29^, ^30^, ^31^, ^32^, ^33^ The resources can be used by multiple key groups to inform decision-making based on what is driving them to collect quality-of-care data. Specifically, regulators often focus on quality assurance and compliance to avoid adverse events, whereas practices and clinicians may emphasize aspects of quality improvement (eg, clinical safety, patient satisfaction, and efficiency), and patients prioritize provider selection or patient-centeredness.^34^ Thus, although many of the resources below define and list quality measures, these primary drivers will determine how each key group uses the resource.

**Table 1:** List of quality-of-care resources with descriptions and website links

Resource	Description	Link
CMS Measure Management System^26^	Provides an overview of measurements	https://mmshub.cms.gov/about-quality/new-to-measures/what-is-a-measure
AHRQ: Types of Healthcare Quality Measures^27^	Provides an overview of types of measurements	https://www.ahrq.gov/talkingquality/measures/types.html
AHRQ: Data sources for Healthcare Quality Measures^28^	Provides an overview of data sources	https://www.ahrq.gov/talkingquality/measures/understand/index.html
AHRQ: Measures of quality for different Health Care Settings^29^	Provides an overview of considerations for different health care settings	https://www.ahrq.gov/talkingquality/measures/setting/index.html
NQF^30^	Not-for-profit organization that develops and implements a national strategy for health care quality measurement and reporting Maintains sets of measures based on a consensus process and adherence to rigorous criteria	https://www.qualityforum.org/Home.aspx
Partnership for Quality Measurement^31^	Provides quality measure consensus review and resources on behalf of CMS	https://p4qm.org/MSR/MSR-resources
CMS Measures Inventory Tool^32^	Measures used by the Centers for Medicare & Medicaid Services in various quality, reporting, and payment programs	https://www.cms.gov/medicare/quality/measures/cms-measures-inventory
Guideline central^33^	Inventory of quality measures created for AHRQ’s measures repository	https://www.guidelinecentral.com/guidelines/

Abbreviations: AHRQ, Agency for Healthcare Research and Quality; NQF, National Quality Forum.

### How is quality related to value?

Quality of care and value-based care are frequently conflated in health care discussions, but they are distinct concepts. Health refers to a person's overall state of physical and mental well-being, whereas care encompasses the services and interventions provided to improve that state. This distinction underpins the evolving dialogue about how we define and measure value in health care. Quality of care relates to the standards and subsequent outcomes of services and interventions delivered, and is usually assessed through the IOM framework detailed above,^16^ with examples of O&P care depicted in figure 1.

In contrast, value-based care is a model of health care delivery that prioritizes achieving better patient outcomes relative to the cost of care.^35^ Therefore, value-based care is not dependent on whether high-quality care was delivered—it depends on whether the care led to meaningful improvements in a patient’s health state reconciled with the cost of that care.^36^ The value of care can be expressed as quality (patient outcomes and experience) divided by the cost of care.^37^

To operationalize value-based care, quality measurement should not only assess the care process but also align with outcomes that matter to patients and costs that reflect equitable and sustainable care. Examples of value-based care strategies include offering preventive services, enabling early detection of chronic conditions, improving care coordination, and reducing avoidable emergency department visits—all of which must be evaluated not just on process quality, but on their ability to improve health at a sustainable cost to the system.^36^

### Understanding terminology

The broader health care community emphasizes precise definitions to avoid miscommunication about quality improvement practices.^38^^,^^39^ Thus, definitions are essential: (1) Quality Measures are explicit and quantifiable items, such as patient satisfaction scores or readmission rates. They directly assess aspects of care delivery; (2) Quality Indicators set minimum acceptable performance standards or benchmarks for practice; and (3) Quality Metrics describe broader system performance indicators (eg, overall hospital efficiency), although used interchangeably with “measures” in literature because of inconsistent usage across contexts.^28^

Because of inconsistencies in the health care literature, terms like “metrics” and “measures” are used interchangeably. For clarity, this article adopts “measures” to represent specific, quantifiable assessments directly tied to quality improvement practices.

Differences between quality measures and indicators are important to note. For example, a quality measure could be that a fall risk assessment should occur within 3 months after the date of prosthetic delivery. A resulting quality indicator could be the percentage of patients who were assessed for risk of falling within 3 months after the date of prosthetic delivery.

## Implications for O&P

Quality measurement is critical as health care systems transition toward value-based care. In 2022, the Centers for Medicare and Medicaid Services (CMS) initiated a National Quality Strategy aiming for a full shift toward alternative payment models in alignment with value-based care by 2030.^40^ This shift emphasizes alignment of reimbursement to health care providers to encourage and reward high-quality, high-value care rather than quantity of services provided. Consequently, the O&P community should begin assessing and prioritizing quality-of-care measures.

Implementing robust quality measures and programs specific to O&P positions O&P clinicians as essential contributors to the health care delivery chain and helps ensure that patients continue to have access to vital O&P interventions. Accurate and specific O&P quality measurement is key, as it will lead to the creation of O&P-specific quality indicators, which can facilitate targeted quality improvement initiatives within O&P care.

Quality-of-care measurement and constant improvement align with the goals and priorities established in the 2017 CMS Meaningful Measures Initiative.^41^ This initiative emphasizes the necessity of quality measures in driving improvements within health care, shaping an “ecosystem of quality measures” essential to value-based care implementation.^40^ CMS’ National Quality Strategy outlines specific pillars—including items such as engagement, resiliency, alignment, interoperability, and scientific advancement—that provide a framework for actionable quality measurement in O&P practice.

Given these developments, O&P practices should consider adopting comprehensive quality measurement practices, thereby positioning themselves in the evolving health care landscape and ensuring continued patient access to essential care.

### Learning from other health care professions to establish quality of care specific to O&P

Quality measures selected for the O&P profession are essential to achieving meaningful care and quality improvements. Although national standards for quality-of-care measures and indicators do not exist specifically for O&P practice, current standards from similar patient populations offer valuable starting points. For example, a quality measure for individuals undergoing hip and knee joint replacements is patient understanding of discharge instructions, a factor directly relatable to patient comprehension of device instructions in O&P practice.^42^ In addition, quality-of-care in musculoskeletal conditions and quality indicators for older adults (eg, annual medical device inspections, providing comprehensive rehabilitation process education, and involving caregivers during treatment sessions) could apply to O&P care.^43^^,^^44^ Recent literature also highlights how quality-of-care measures can be developed and implemented effectively in outpatient settings, notably within PT, which closely parallels O&P practice.^45^ The American Physical Therapy Association's outcome registry offers a valuable model for standardizing quality measures, providing a template that O&P organizations could adopt for promoting consistent quality-of-care measurement and improvement.^46^ This registry emphasizes the use of validated tools that align with clinical practice guidelines and the International Classification of Functioning, Disability and Health framework.^47^

Although still evolving, the Limb Loss and Preservation Registry is a potentially powerful tool for the O&P profession.^48^ Launched in 2022, the Registry continues to expand to meet the growing need for comprehensive, quality-of-care data. It systematically collects, analyzes, and reports standardized information on individuals with limb loss or those at risk of limb loss. The data are contributed nationwide by hospitals and O&P clinics. If hospitals and O&P patient care organizations mobilize to contribute broadly, the registry can represent more fully the patient population. If the O&P field develops and incorporates standardized quality-of-care indicators into the Registry’s data and reporting framework, it could become a powerful driver of improved clinical outcomes, best-practice guidance, and evidence-based decision-making across the continuum of O&P care.

Sustainable quality improvement initiatives are crucial to effectively implementing quality measures in O&P practice, and typically hinge upon 6 key implementation components: accountability, education, communication, monitoring and reporting, structure, and processes.^49^^,^^50^ These principles, applied across various health care domains, including inpatient nursing and joint replacement care, can guide infrastructure development for O&P practices.^51^ Current processes within O&P practice reflect foundational aspects of quality care, providing useful entry points for standardization. For instance, the Food and Drug Administration’s regulation of cranial remolding orthoses reflects the importance of standardized practice for device safety and effectiveness.^52^ Similarly, organizational standardization for patient documentation, component ordering, and clinician performance provides frameworks readily adaptable for rigorous quality-of-care measure collection and storage. The growing use of technological advancements, such as 3D printing, further necessitates enhanced standardization and quality-of-care measure integration.

O&P practices are focused on evidence-based care to improve coverage and treatment. To boost clinical results and operations, O&P must also start using micro-level data such as practitioner-specific quality scores for decision-making. Collecting this specific quality data allows for continuous improvement, which is essential for O&P to engage with health care partners shifting to value-based care. Therefore, quality-of-care education and research must be a priority across all O&P training programs.

## Key groups moving towards measuring quality of care

The frameworks and models discussed in this special communication are rarely discussed in the O&P field. An enhanced understanding by orthotists and prosthetists of quality-of-care terminology, frameworks, and models is essential for promoting interdisciplinary communication across health care disciplines. This section outlines potential responsibilities for each key group in O&P, building on experiences documented in related health care professions.

### Leaders in O&P organizations: facilitate awareness and standardization of quality of care

Leaders in O&P organizations, both within for-profit and not-for-profit, can raise awareness of quality of care among O&P professionals by offering workshops or webinars. In addition, these organizations can create working groups to lead national discussions on quality of care and standardization for data collection and distribution. For example, a working group could establish common data elements, similar to the National Institute of Neurological Disorders and Stroke NeuroRehab working group, which provides a standard data set for neurorehabilitation research.^53^ O&P organizations can also partner with registries to collaboratively implement national standardized shifts toward quality of care. Nonprofit organizations within O&P that share a common vision for advancing O&P care through funding for clinical research can establish quality of care as a focus for research within the profession. Accrediting organizations such as the American Board for Certification in Orthotics, Prosthetics and Pedorthics, the Board of Certification/Accreditation, and the Commission on Accreditation on Rehabilitation of Facilities International also value evidence-based practice and quality reporting.

### Researchers: establish quality measures and quality indicators

Researchers in O&P can use the body of literature in PT, OT, and nursing professions as a guide to identify broader patient and clinician priorities, program measures, and to initiate quality-of-care studies from a management perspective.^54^, ^55^, ^56^, ^57^, ^58^, ^59^, ^60^ After the establishment of quality measures, quality indicators can be defined from clinical practice guidelines; however, few guidelines exist in O&P. In the absence of established clinical practice or operational guidelines, consensus-based methods such as Delphi expert panels, along with systematic literature reviews, can guide indicator development.^60^, ^61^ Frameworks—such as the 6 aims for health care systems (safe, effective, patient-centered, timely, efficient, and equitable)—can inform the development of qualitative interviews and surveys to prioritize quality-of-care elements.^15^^,^^16^ To support the rigorous research needed to establish universal quality-of-care measures, the O&P profession should cultivate researchers who can assess the quality of O&P services, emulating research frameworks used in PT and OT.

### Manufacturers: report device safety and effectiveness

O&P manufacturers can contribute to high-quality care by evaluating and communicating device safety and effectiveness to clinicians, patients, and clinic owners. For example, surgical instruments that have inadequate quality control (eg, high levels of contamination) result in higher odds of postsurgical infections, despite efforts by hospitals and surgeons to maintain a sterile environment. Similarly, an O&P clinic owner and clinician can ensure device safety regarding prosthetic alignment but rely on the O&P manufacturer’s testing of componentry (eg, microprocessor knee) to prevent falls, or orthotic joints manufactured to appropriate International Organization for Standardization levels to prevent early device failure that could cause harm. Manufacturers can be more transparent about quality control processes and procedures to ensure documented high quality in the O&P care delivery chain, which contributes to high-value care through better patient outcomes.

O&P manufacturers in the US are increasingly buying patient care practices and delivering services directly. This integration, however, makes their quality of care vulnerable, because issues can affect both manufacturing and clinical care. Despite these risks, manufacturers have an advantage: covering the entire process—from developing and building devices to fitting and delivering them to patients. Their substantial financial and personnel resources position them well to manage the challenges of the shift to value-based care and even excel within it. Manufacturers with patient care operations must lead this industry transition. They have a vested interest in a stable market and should not want to see independent O&P clinics or clinicians fail. Therefore, they should share their experience across the industry. Furthermore, these organizations could assess care quality (safety, effectiveness, patient-centeredness, timeliness, and efficiency) across their practices, aligning with recommendations for all clinic owners.

### Clinic owners: implementing quality programs

Once quality measures and indicators have been selected, O&P practices can establish structures, procedures, and systems for the longitudinal collection and tracking of these measures, as well as for public reporting. Common barriers to implementing quality indicators, as identified by physical therapists, include insufficient knowledge, limited time, and lack of management support.^55^ Quality improvement initiatives, targeted training, and educational programs can mitigate these barriers. For example, data analysts or collectors could be integrated into management teams across multiple practices, creating standardized benchmarks for ongoing quality improvements—especially valuable as industry consolidation increases. Quality improvement initiatives can include compliance aspects, which many clinics already implement, that are central to ensuring ethical clinical practice. Academic partnerships may facilitate the development of robust quality improvement initiatives.^62^ The Institute for Healthcare Improvement offers free resources on quality improvement, which clinic owners could use to guide quality improvement initiatives.^63^

O&P practices may participate or be forced into alternative payment models through Accountable Care Organizations or Integrated Delivery Networks. By advancing research on quality improvement and demonstrating the value of O&P within the overall care delivery chain, the profession can shape its value proposition and ensure its critical contributions are recognized by all key groups in the health care system. Thus, research on quality improvement and implementation science, along with learning health system methodologies,^64^ will be critical for evaluating strategies to collect and track quality measures effectively within O&P practices.

### Clinicians: collect patient care quality measures

When quality-of-care measures and indicators are established, researchers and clinicians have support from clinic owners; clinicians can collect relevant information from patients. Clinicians can recognize that quality-of-care measurement encompasses broad dimensions beyond simply clinical outcomes, quality of life, or patient satisfaction. O&P professionals can use the terms “clinical outcome measures” (eg, an instrument to measure a dimension, such as quality of life) and “outcomes” (eg, a final result, such as hospital readmission or mortality rates) appropriately to align with established terminology in the broader health care community.^35^^,^^36^

For instance, care team members could collect patient-reported data while the patient is in the waiting room before an appointment, which could help inform the direction of the appointment and clinical decision-making and be stored within the outcomes domain of the patient’s medical record. Clinicians can use this information to inform clinical actions and decision-making, in addition to reporting purposes. Examples include the Prosthetic Limb Users Survey of Mobility for measuring mobility, the Orthotic Prosthetic Users Survey for measuring function, quality of life, and satisfaction with services and devices, or the Patient-Reported Outcome Measurement Information System for assessing quality of life.^65^, ^66^, ^67^, ^68^ Figure 1 lists other examples that could be collected. It is important for clinicians to participate in research to establish quality-of-care measures and indicators, along with promoting patient participation in quality of care within their clinics, with the support of clinic owners.

### Patients: consider quality-of-care data in provider selection

Implementation and transparent reporting of quality measures by key groups, patients can make informed decisions regarding their O&P care. Specifically, if patients were equipped with this information, they could use quality-of-care data to aid in provider selection, increasing their autonomy and increasing patient engagement. Although additional effort can be required to switch or select O&P clinicians, this approach aligns with current hospital standards following CMS mandates that quality-of-care data be publicly reported.^69^^,^^70^ If patients request quality-of-care data, such as satisfaction scores and patient outcomes, it is more likely that clinics and clinicians will prioritize the systematic collection and distribution of this information. Finally, patients can participate in quality-of-care research studies to establish meaningful quality-of-care priorities and measures.

### Educators: incorporate quality into the curriculum

To contribute to expert panels and research activities aimed at establishing quality-of-care measures and indicators, orthotists and prosthetists must possess an understanding of quality-of-care concepts, as well as value-based care and alternative payment models. Recommendations from other health care professions emphasize the importance of integrating value-based care principles within curricula to prepare graduates for practice demands.^71^ Consequently, O&P educational programs should incorporate coursework focused on quality-of-care frameworks, the fundamentals of value-based care, and the practical implementation of quality measures. Incorporating quality-of-care training into graduate curricula is imperative for preparing clinicians and researchers capable of leading this critical transition.

## Future directions

**Fig 2:**
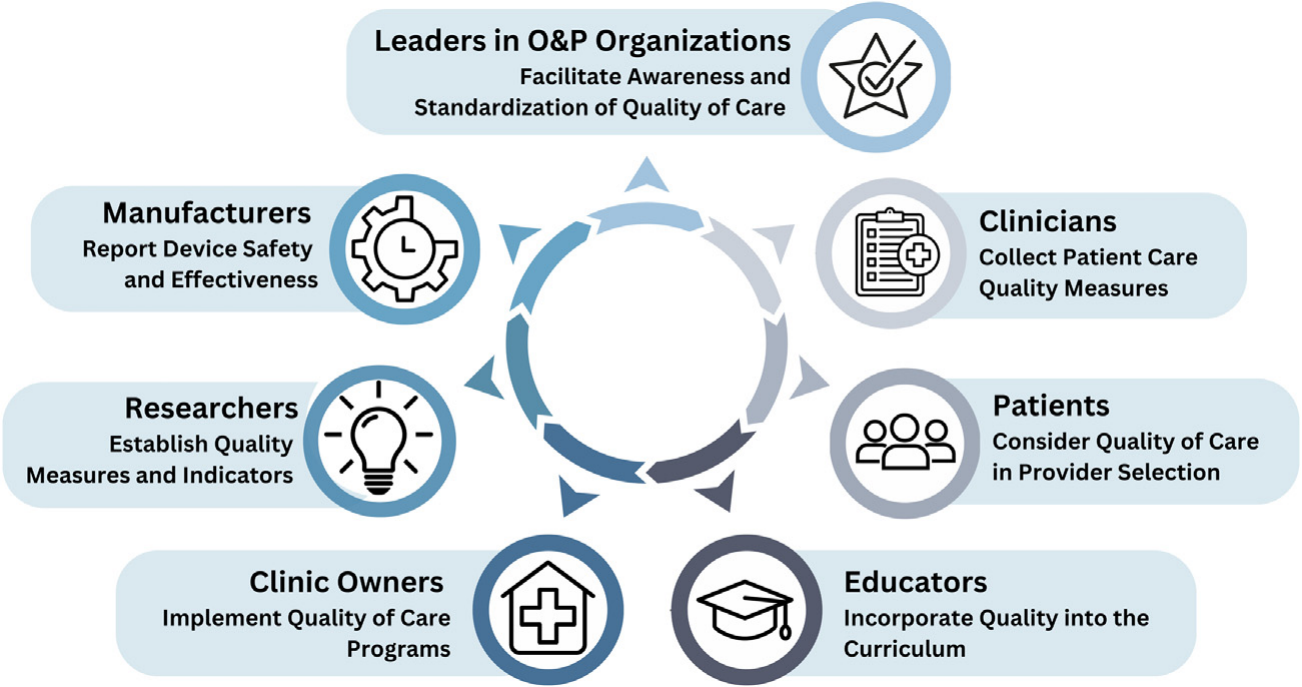
Summary of key groups and main roles to promote quality of care in O&P practice.

Through key action items (fig 2; table 2), the O&P profession can establish specific quality-of-care measures and reporting practices to improve patient outcomes, allow more patients to benefit from O&P rehabilitation, and efficiently transition toward a reimbursement model based on quality metrics. Each key group will play a vital role in advancing the O&P field toward a quality-based, patient-centered care model, ensuring improved patient outcomes and long-term sustainability of the profession.

**Table 2:** Summary of action items for each key group

Group	Action Items
Leaders in O&P organizations: facilitate awareness and standardization of quality of care	• Raise awareness of quality of care to O&P professionals through providing organizational resources • Establish working groups on quality of care • Partner with registries and appropriate nonprofit organizations focused on research and education to support quality of care priorities
Researchers: establish quality measures and quality indicators	• Identify measures to inform indicators from patient and clinician perspectives, expert panels, or consensus studies • Adopt frameworks and terminology for quality of care • Use implementation science to evaluate systems and programs • Seek out training and collaboration to produce research in this space
Manufacturers: report device safety and effectiveness	• Collect and report quality of care to demonstrate safety and effectiveness of devices • Report quality of control process and procedures • Widen quality-of-care measures to patient-centeredness, equity, and efficiency as the scope of the manufacturing company widens to patient care delivery
Clinic owners: implement quality-of-care programs	• Develop quality programs at the institutional and clinical levels to build out the structure and process components of implementation and accountability for training programs and continuous improvement initiatives • Create a sustainable environment in practice for collecting measures (eg, time to delivery, also patient satisfaction) and benchmarks for quality improvement over time • Partner with researchers/academics to help facilitate quality-of-care research
Clinicians: collect patient care quality measures	• Understand the importance of quality of care, why and how it is measured • Professional development for continual process and quality improvement • Partner with researchers/academics to help facilitate research-learning health systems
Patients: consider quality of care in provider selection	• Consider what components of quality of care are most important to you • Participate in quality-of-care research • Engage in discussions to incorporate quality improvement into scope of practice and training standards • Ask clinics and clinicians about quality-of-care components, such as patient satisfaction rates
Educators: incorporate quality into the curriculum	• Present frameworks and aspects of quality of care • Discuss the relevance and impending translation of quality of care to the O&P field • Frame quality improvement as a professional responsibility

## Disclosures

The investigators have no financial or nonfinancial disclosures to make in relation to this project.
